# Cross‐sectional area on magnetic resonance images of the semitendinosus tendon is strongly related to the collagen fibril diameter

**DOI:** 10.1002/jeo2.70124

**Published:** 2024-12-30

**Authors:** Yushin Mizuno, Junsuke Nakase, Tatsuya Ishikawa, Kazuki Asai, Yasushi Takata, Tomoyuki Kanayama, Takuya Sengoku, Noriyuki Ozaki, Hiroyuki Tsuchiya

**Affiliations:** ^1^ Department of Orthopaedic Surgery Graduate School of Medical Sciences Kanazawa University Kanazawa Ishikawa Japan; ^2^ Section of Rehabilitation Kanazawa University Hospital Kanazawa Ishikawa Japan; ^3^ Department of Functional Anatomy Graduate School of Medical Sciences Kanazawa University Kanazawa Ishikawa Japan; ^4^ Department of Orthopaedic Surgery Keiju Medical Center Nanao Ishikawa Japan; ^5^ Department of Orthopaedic Surgery KKR Hokuriku Hospital Kanazawa Ishikawa Japan; ^6^ Department of Orthopaedic Surgery Yokohama Sakae Kyosai Hospital Yokohama Kanagawa Japan

**Keywords:** anterior cruciate ligament reconstruction, collagen fibril diameter, cross‐sectional area, graft failure, semitendinosus tendon

## Abstract

**Purpose:**

Using a thin semitendinosus tendon as an autograft is a risk factor for poor clinical outcomes after anterior cruciate ligament reconstruction. Preoperative evaluation of the cross‐sectional area of the semitendinosus tendon using magnetic resonance imaging is useful. However, studies comparing the cross‐sectional area of the semitendinosus tendon on magnetic resonance imaging and the collagen fibril diameter of the semitendinosus tendon are lacking. We aimed to investigate the relationship between collagen fibril diameter and cross‐sectional area of the semitendinosus tendon using magnetic resonance imaging.

**Methods:**

We included 14 patients (24.5 ± 12.3 years) who underwent anterior cruciate or medial patellofemoral ligament reconstruction using the semitendinosus tendon. Samples not used to prepare autografts were used to evaluate the collagen fibril diameter. Transmission electron microscopy was used to measure several hundred short fibril diameters per sample. Magnetic resonance imaging (T2‐weighted imaging) was used to assess the cross‐sectional area of the semitendinosus tendon, measured 8 cm proximal to the tibial attachment. Spearman's rank correlation coefficients were determined for collagen fibril diameter and cross‐sectional area of the semitendinosus tendon on magnetic resonance imaging, and the relationship between both parameters was evaluated.

**Results:**

The collagen fibril diameter of the semitendinosus tendon was calculated from 10,279 fibrils. The correlation coefficient between the collagen fibril diameter and the cross‐sectional area of the semitendinosus tendon was 0.821 (*p* < 0.001).

**Conclusions:**

A strong positive correlation was observed between the collagen fibril diameter and cross‐sectional area of the semitendinosus tendon. A small cross‐sectional area on the magnetic resonance image of the semitendinosus tendon indicated a thin collagen fibril diameter, which may affect the mechanical strength of the autograft for anterior cruciate ligament reconstruction. The collagen fibril diameter can be predicted preoperatively by measuring the cross‐sectional area of the semitendinosus tendon using magnetic resonance imaging.

**Level of Evidence:**

Level IV.

**Abbreviations**
ACL: anterior cruciate ligament
ICC: intraclass correlation coefficient
MRI: magnetic resonance imaging
ST: semitendinosus tendon

## INTRODUCTION

Anterior cruciate ligament (ACL) injuries are among the most frequent sports knee injuries, and reconstruction is the gold standard of treatment because natural healing of the injuries is complicated [7, 20]. Among the several options for ACL reconstruction [13, 16, 27], the semitendinosus tendon (ST) is frequently used owing to its ease of harvesting and minimal inconvenience after harvesting [15, 22, 23]. Re‐operation, which is the most significant postoperative concern, is more likely to occur after reconstruction with the ST as the autograft [4, 18, 21].

Recent studies have focused on the microstructure of ST, revealing that the collagen fibril diameter of the ST increases with skeletal maturity [1] and that smaller collagen fibril diameter of the ST correlates with greater strain at maximum stress and smaller mechanical strength [2]. In other words, STs with larger collagen fibril diameters may function as mechanically stronger autografts. However, actual measurement of the collagen fibril diameter of the ST preoperatively is impossible.

Therefore, we focused on the relationship between the cross‐sectional area on preoperative magnetic resonance imaging (MRI) and the collagen fibril diameter of the ST. Clarification of this relationship will allow the preoperative prediction of the collagen fibril diameter of the ST, which will be useful in selecting autografts to reduce re‐operation rates; in addition, the clarification will have important and direct clinical implications. We hypothesised that the cross‐sectional area of the ST on MRI is positively correlated with the collagen fibril diameter of the ST. Therefore, this study aimed to determine this relationship and validate our hypotheses.

## MATERIALS AND METHODS

This study was conducted with the approval of the Medical Ethics Review Committee of our institution (Approval Number: 2017‐324) and in accordance with the Declaration of Helsinki. Patients were informed about the study verbally and in writing, and they provided their consent to participate. The same explanation was provided to the parents of minors. Minors were defined as patients below 20 years of age at the time the patients underwent surgery.

### Patient characteristics and sample collection

We included 18 patients who underwent ACL or medial patellofemoral ligament reconstruction using a hamstring tendon autograft between August 2018 and June 2019. The patients did not have a history of trauma or surgery around the knee. We excluded four patients whose preoperative MRI of the axial section did not include an image at a position 8 cm proximal to the ST attachment site. This position coincided with the position where the cross‐sectional area of the ST was measured, which will be explained in the ‘Measurement of cross‐sectional area of the ST’ section. Finally, 14 patients (seven men and seven women; age, 24.5 ± 12.3 years [mean ± standard deviation]; height, 165.3 ± 8.4 cm; weight, 58.4 ± 7.7 kg) were included in the study.

**Figure 1:**
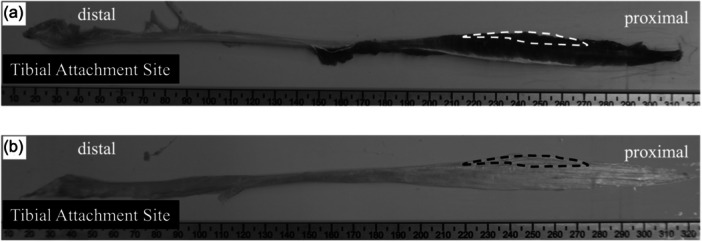
Collection site of the semitendinosus tendon. Samples were obtained from the myotendinous transition zone (dotted enclosure) of the semitendinosus muscle. (a) Semitendinosus muscle immediately after harvesting for autograft creation. (b) Semitendinosus tendon after the muscle shaving process.

Samples for microstructural analysis using transmission electron microscopy were obtained from the myotendinous transition zone of the semitendinosus muscle (Figure 1a,b). Notably, this zone was removed during the trimming process to create the graft for reconstruction and was no longer required.

### Measurement of collagen fibril diameter of the ST

**Figure 2:**
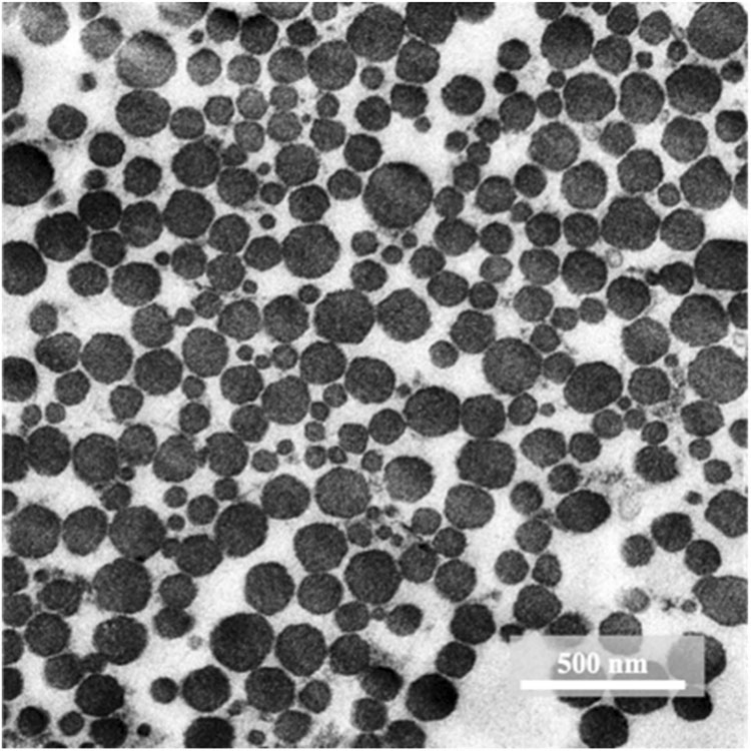
Measurement of collagen fibril diameter in the semitendinosus tendon using transmission electron microscopy. The smallest diameter of each fibril was measured to assess the correct fibril diameter of collagen in tendons.

The tendon tissues were fixed overnight in 2% paraformaldehyde with 2.5% glutaraldehyde in 0.1 M phosphate buffer (pH 7.4) at 4°C, then incubated in a fixative agent containing 2% osmium tetroxide for 60 min on ice and counterstained en bloc with 1% uranyl acetate overnight at 4°C. The tendons were dehydrated using sequential treatment for 10 min in 50%, 70%, 80%, 90%, 95% and 100% ethanol and placed in a second solution of 100% ethanol before being incubated in n‐butyl glycidyl ether twice. These samples were incubated in a 1:1 mixture of QY‐1/Quetol‐812 resin for 10 min and infiltrated overnight with Quetol‐812. After resin curing at 60°C for 2 days, ultrathin sections (70 nm thick) were prepared using an ultramicrotome (Ultracut‐T, Leica, Germany or 8800 ULTROTOME III, LKB). Subsequently, the sections were placed on copper grids and contrasted with lead citrate for 5 min. The samples were examined at ×8000 magnification under a transmission electron microscope (H‐7650, Hitachi). Digital electron micrographs of ultrathin sections were then captured (Figure 2).

Minimum collagen fibril diameters were measured from these cross‐sections using ImageJ software (US National Institutes of Health). Notably, when the collagen fibrils of tendons are cut obliquely and not cross‐sectionally, they exhibit an elliptical morphology owing to the cylindrical shape of the fibrils [8, 10]. Therefore, the minimum diameter of each fibril can be used to estimate the actual fibril diameter. Furthermore, the measurements did not include any fibrils exhibiting longitudinal profiles with cross‐striated banding patterns. A minimum of four sections and 300 collagen fibrils from each sample were evaluated, and the average was used as the representative value.

### Measurement of cross‐sectional area of the ST

The participants underwent MRI using an eight‐channel knee coil in the supine position, with the knee slightly flexed. Two MRI systems (Ingenia CX, Philips Healthcare; AIRIS Vento Plus, FUJIFILM Healthcare Systems Corporation) were used. The magnetic field strengths of these devices were 1.5 T and 0.3 T, respectively. The reason for the different T values of the MRIs used is that, in some cases, images were taken at nearby medical facilities owing to an overflow of patients. Twelve patients were imaged at 1.5 T, and only two patients were imaged at 0.3 T. The basic imaging conditions were as follows: imaging sequence, T2‐weighted fast spin‐echo; field of view, 150 × 150 mm; repetition time, 4000 ms; echo time, 100 ms; slice thickness, 4 mm; slice gap, 1 mm and number of signals averaged, 2.

**Figure 3:**
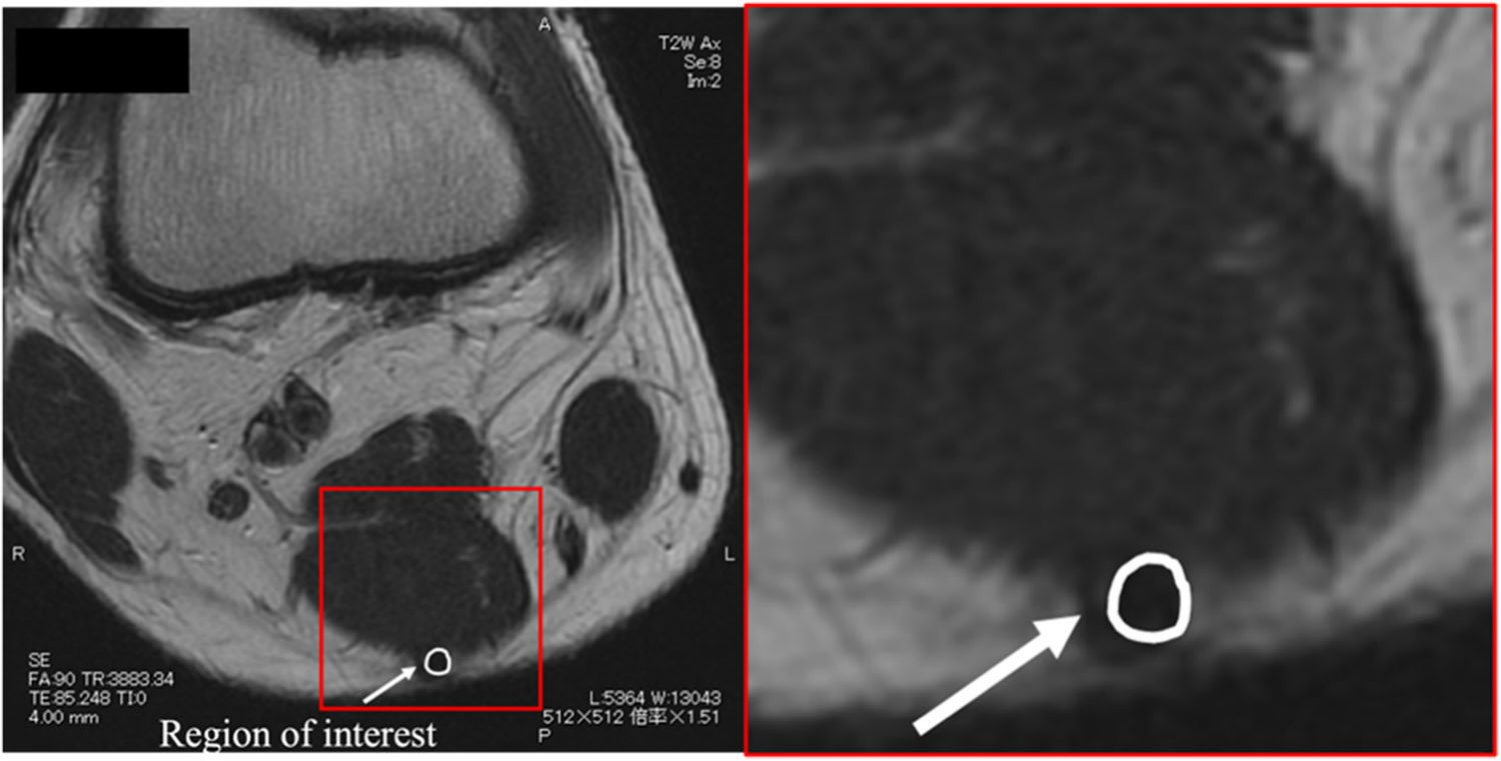
Measurement of the cross‐sectional area of the semitendinosus tendon using magnetic resonance imaging T2 weighted images. Measurements were calculated using a polygonal region of interest at a position 8 cm proximal to the tibial attachment of the semitendinosus tendon.

Cross‐sectional area of the ST was measured using MRI with this technique. Although previous studies measured the cross‐sectional area of the ST at various elevations around the knee joint [5, 12, 14], these studies investigated the association between the ST diameter obtained intraoperatively and that observed on MRI, which differed from the objective of the present study. Therefore, we considered the graft preparation procedure and took measurements at a position 8 cm proximal to the axial section from the ST attachment site, the pes anserinus. This location corresponds to the central length of the ST autograft. The obtained images were magnified as much as possible, and the signal difference between the tendon and muscle of the ST was used to distinguish between the two [11, 12]. The cross‐sectional area of the ST was measured using polygonal regions of interest (Figure 3). This distinction included a thorough review by an orthopaedic surgeon and a physical therapist familiar with musculoskeletal structures.

### Statistical analysis

Before evaluating the relationship between the cross‐sectional area on preoperative MRI and the collagen fibril diameter of the ST, normality of the respective data was confirmed using the Shapiro–Wilk test. Subsequently, Spearman's rank correlation coefficient was calculated to evaluate the relationship between the collagen fibril diameter and the cross‐sectional area of the ST. We used SPSS Statistics version 29.0 (IBM Corp.) for statistical analysis. The significance level was set at *p* < 0.05.

## RESULTS

First, to check the reliability of the measurement for the cross‐sectional area of the ST obtained via our method, the intraclass correlation coefficient (ICC) was calculated, and the intra‐ (1, 2) and interrater (2, 1) reliabilities were investigated. The results revealed that ICC (1, 2) and ICC (2, 1) were 0.897 and 0.703, respectively, and ICC (1, 2) calculations were performed after a 3‐day blank period. Fourteen data sets were used for the calculations.

**Figure 4:**
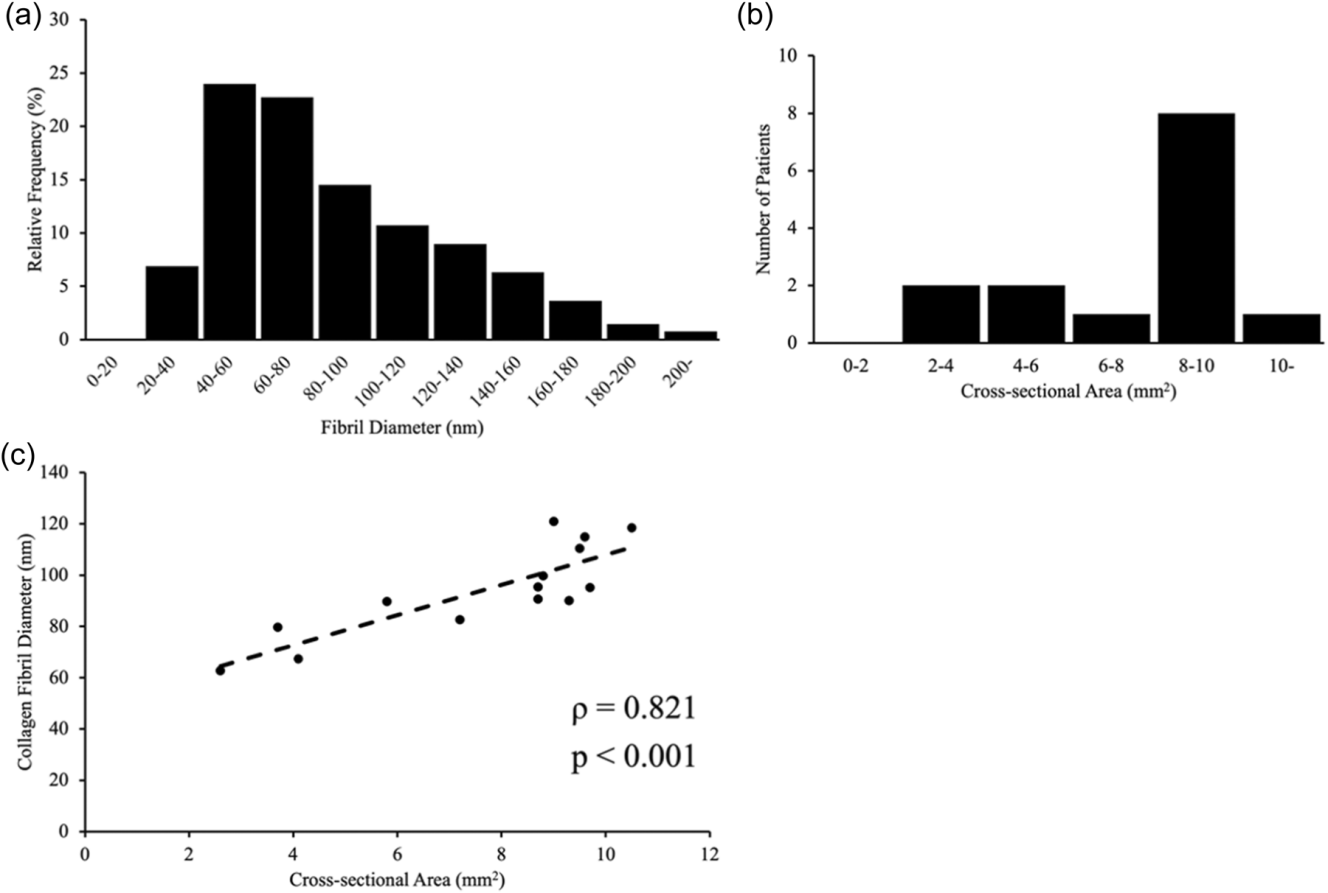
Distribution of all data and their correlations. The collagen fibril diameter was calculated from 10,279 fibrils (a). Transmission electron microscopy was used for the measurements. The cross‐sectional area of the semitendinosus tendon measured 8 cm proximal to the attachment (b). The Spearman's rank correlation coefficient was 0.821 (c).

The number of collagen fibril diameters in the ST that could be measured per patient was 734.2 ± 316, leading to a total of 10,279. The mean collagen fibril diameter was 94.1 ± 17.8 nm (Figure 4a), and the median cross‐sectional area was 8.75 (minimum: 2.60, maximum: 10.5) mm^2^ (Figure 4b). The collagen fibril diameter data showed normality (*p* ≥ 0.05); however, the cross‐sectional area data did not show normality (*p* < 0.05). Finally, a strong positive correlation (*p* = 0.821, 95% confidence interval: 0.501–0.943) was found between the cross‐sectional area and collagen fibril diameter of the ST (*p *< 0.001; Figure 4c).

Based on these results, a post hoc power analysis was performed using G*Power 3.1 (Franz Paul) [9]. Assuming a two‐tailed test for the population correlation coefficient with a correlation coefficient of 0.843, a significance level of 5%, and a sample size of 14 cases, the power was 0.999.

## DISCUSSION

This study found that the cross‐sectional area of the ST on MRI is strongly related to the diameter of its microstructure, that is, collagen fibrils. To the best of our knowledge, this study is the first to report the relationship between the diameter of the tendon on MRI and that of collagen fibrils, which are the main component of the tendons.

The collagen fibre diameter of the ST has been a target of research in recent years. Asai et al. [1] collected ST tissues from 18 patients and classified them into immature (<20 years old, epiphyseal line >1.5 mm), young (<20 years old, epiphyseal line <1.5 mm), and adult groups (>20 years old) based on patient age and the amount of patency of the epiphyseal line and investigated age‐related changes in the collagen fibril diameter. The collagen fibril diameter of the ST was measured in each group, and the fibril diameter of the immature group (73.1 ± 10.5 nm) was significantly (*p* < 0.01) smaller than that of the adult group (105.8 ± 14.9 nm). In addition, Asai et al. [2] investigated the relationship between the collagen fibril diameter and mechanical strength (strain and stress) of the ST using 22 samples. They found that the collagen fibril diameter showed a significant (*p* < 0.01) negative correlation with strain (*r* = –0.596) and a significant (*p* < 0.05) positive correlation with stress (*r* = 0.484). Notably, tendon tissues of the same size were used for this mechanical evaluation. Their evaluation in the previous study was performed in a blinded manner. Incidentally, the relationship between collagen fibril diameter and mechanical strength in tendon tissue has also been confirmed in tissues derived from mice [6]. Taken together, ST with a large collagen fibril diameter may function as a mechanically strong autograft for ACL reconstruction. Furthermore, the results of the present study suggest that superior ST autografts have a larger cross‐sectional area on MRI. However, collagen fibrils in tendon tissue cannot be evaluated using transmission electron microscopy before reconstruction. Therefore, the results of this study suggest that the cross‐sectional area of the ST on MRI may be used to preoperatively estimate the collagen fibril diameter of the ST, as well as to identify and exclude patients whose ST is not suitable for use as autografts.

The relationship between the cross‐sectional area and collagen fibril diameter of the ST remains unclear. STs with a diameter ≤8 mm, a component of the cross‐sectional area, are more likely to cause re‐injury postoperatively [24, 26]. In the present study, we demonstrated a positive correlation between the cross‐sectional area and collagen fibril diameter of the ST. Given that the collagen fibril diameter of the ST is closely related to mechanical strength, as described above [2], the association between the small diameter of the ST and the incidence of re‐injury may indirectly reflect the results of this study.

One strength of this study is that we investigated the relationship between the cross‐sectional area and the collagen fibril diameter using the ST obtained from humans rather than that from animals. In addition, the results of this study provide clinicians with evidence for the possibility of estimating the microstructure of the ST from preoperative MRI. However, this study had some limitations. First, the samples from which the fibril diameters were evaluated were taken from the outside of the autografts. In addition, this study used collagen fibril diameter in the ST at the time of reconstruction as the value of interest and did not examine the ligamentization process occurring postoperatively. Therefore, we could not directly evaluate the autografts in ACL reconstruction or reconstructed ligaments after reconstruction. However, this limitation does not seem to be avoidable from an ethical perspective. Second, we did not perform any direct mechanical testing of the samples. Nevertheless, a relationship between the fibril diameter and mechanical strength has previously been reported [2, 6]. Third, owing to the small sample size, we could not examine the association between cross‐sectional area and collagen fibril diameter based on age or sex. Female sex and younger age are considered risk factors for re‐injury after ACL reconstruction; [3, 19, 25] therefore, these details will provide useful additional information. Finally, the relationship between the actual collagen fibril diameter in the ST and the re‐operation rate remains unclear, and thus, requires further investigation. Re‐operation after ACL reconstruction is associated with various risk factors [17]. Additional studies on the relationship between cross‐sectional area and collagen fibril diameter of the ST, as revealed in this study, will clarify their association with re‐operation rates.

## CONCLUSIONS

This study aimed to clarify the relationship between the cross‐sectional area of the ST on MRI and the collagen fibril diameter on transmission electron microscopy of the ST; the results showed a strong positive correlation between the two parameters. These results suggest that the collagen fibril diameter can be predicted preoperatively by measuring the cross‐sectional area of the ST using MRI.

## AUTHOR CONTRIBUTIONS

Junsuke Nakase conceived the study; Junsuke Nakase, Noriyuki Ozaki and Hiroyuki Tsuchiya managed and supervised the project; Yushin Mizuno, Tatsuya Ishikawa, Kazuki Asai, Tomoyuki Kanayama and Takuya Sengoku conducted the research and analysis; Yushin Mizuno wrote the manuscript; and Kazuki Asai, Yasushi Takata, Tomoyuki Kanayama and Takuya Sengoku reviewed and edited the manuscript. All authors have read and approved the final manuscript.

## CONFLICT OF INTEREST STATEMENT

The authors declare no conflicts of interest.

## ETHICS STATEMENT

This study was conducted with the approval of the Medical Ethics Review Committee of Kanazawa University (2017‐324) and in accordance with the Declaration of Helsinki. Patients were informed about the study verbally and in writing, and consent to participate was obtained from the patients and/or relevant persons (parents/guardians). The study participants were informed of the publication of the study data in academic journals and their consent was obtained. If the participants were minors, approval was obtained from their parents.

## Data Availability

The data underlying this article will be shared by the corresponding author upon reasonable request.

## References

[1] Asai, K. , Nakase, J. , Ishikawa, T. , Yoshimizu, R. , Kimura, M. , Ozaki, N. et al. (2022) Differences in cellular and microstructural properties of the semitendinosus muscle tendon between young and adult patients. Journal of Orthopaedic Science, 27, 478–485. Available from: 10.1016/j.jos.2021.01.012 33714680

[2] Asai, K. , Nakase, J. , Kuzumaki, T. , Ishikawa, T. , Ozaki, N. & Tsuchiya, H. (2023) Differences in the microstructural and mechanical qualities of semitendinosus tendon grafts between skeletally immature and mature patients in anterior cruciate ligament reconstruction. Journal of Orthopaedic Science, 29, 1430–1437. Available from: 10.1016/j.jos.2023.11.004 37985294

[3] Asai, K. , Nakase, J. , Shimozaki, K. , Yoshimizu, R. , Kimura, M. & Tsuchiya, H. (2021) Skeletally immature patient showed lower graft maturity than skeletally mature patient after ACL reconstruction with a rounded rectangular femoral tunnel. Scientific Reports, 11, 19968. Available from: 10.1038/s41598-021-99532-1. 34620936 PMC8497465

[4] Belk, J.W. , Kraeutler, M.J. , Marshall, H.A. , Goodrich, J.A. & Mccarty, E.C. (2018) Quadriceps tendon autograft for primary anterior cruciate ligament reconstruction: a systematic review of comparative studies with minimum 2‐year follow‐up. Arthroscopy: The Journal of Arthroscopic & Related Surgery, 34, 1699–1707. Available from: 10.1016/j.arthro.2018.01.047 29628379

[5] Bickel, B.A. , Fowler, T.T. , Mowbray, J.G. , Adler, B. , Klingele, K. & Phillips, G. (2008) Preoperative magnetic resonance imaging cross‐sectional area for the measurement of hamstring autograft diameter for reconstruction of the adolescent anterior cruciate ligament. Arthroscopy: The Journal of Arthroscopic & Related Surgery, 24, 1336–1341. Available from: 10.1016/j.arthro.2008.07.012 19038703

[6] Connizzo, B.K. , Sarver, J.J. , Iozzo, R.V. , Birk, D.E. & Soslowsky, L.J. (2013) Effect of age and proteoglycan deficiency on collagen fiber re‐alignment and mechanical properties in mouse supraspinatus tendon. Journal of Biomechanical Engineering, 135, 021019. Available from: 10.1115/1.4023234. 23445064 PMC5413158

[7] Cristiani, R. , Mikkelsen, C. , Forssblad, M. , Engström, B. & Stålman, A. (2019) Only one patient out of five achieves symmetrical knee function 6 months after primary anterior cruciate ligament reconstruction. Knee Surgery, Sports Traumatology, Arthroscopy, 27, 3461–3470. Available from: 10.1007/s00167-019-05396-4 30778627 PMC6800857

[8] Dressler, M.R. , Butler, D.L. , Wenstrup, R. , Awad, H.A. , Smith, F. & Boivin, G.P. (2002) A potential mechanism for age‐related declines in patellar tendon biomechanics. Journal of Orthopaedic Research, 20, 1315–1322. Available from: 10.1016/S0736-0266(02)00052-9 12472246

[9] Faul, F. , Erdfelder, E. , Buchner, A. & Lang, A.‐G. (2009) Statistical power analyses using G*Power 3.1: tests for correlation and regression analyses. Behavior Research Methods, 41, 1149–1160. Available from: 10.3758/brm.41.4.1149 19897823

[10] Frank, C. , Bray, D. , Rademaker, A. , Chrusch, C. , Sabiston, P. , Bodie, D. et al. (1989) Electron microscopic quantification of collagen fibril diameters in the rabbit medial collateral ligament: a baseline for comparison. Connective Tissue Research, 19, 11–25. Available from: 10.3109/03008208909016811 2791555

[11] Galanis, N. , Savvidis, M. , Tsifountoudis, I. , Gkouvas, G. , Alafropatis, I. , Kirkos, J. et al. (2016) Correlation between semitendinosus and gracilis tendon cross‐sectional area determined using ultrasound, magnetic resonance imaging and intraoperative tendon measurements. Journal of Electromyography and Kinesiology, 26, 44–51. Available from: 10.1016/j.jelekin.2015.11.006 26708406

[12] Grawe, B.M. , Williams, P.N. , Burge, A. , Voigt, M. , Altchek, D.W. , Hannafin, J.A. et al. (2016) Anterior cruciate ligament reconstruction with autologous hamstring: can preoperative magnetic resonance imaging accurately predict graft diameter? Orthopaedic Journal of Sports Medicine, 4, 2325967116646360. Available from: 10.1177/2325967116646360. 27294166 PMC4887876

[13] Hart, D. , Gurney‐Dunlop, T. , Leiter, J. , Longstaffe, R. , Eid, A.S. , Mcrae, S. et al. (2023) Biomechanics of hamstring tendon, quadriceps tendon, and bone‐patellar tendon‐bone grafts for anterior cruciate ligament reconstruction: a cadaveric study. European Journal of Orthopaedic Surgery & Traumatology, 33, 1067–1074. Available from: 10.1007/s00590-022-03247-6 35362777

[14] Hodges, C.T. , Shelton, T.J. , Bateni, C.P. , Henrichon, S.S. , Skaggs, A.W. , Boutin, R.D. et al. (2019) The medial epicondyle of the distal femur is the optimal location for MRI measurement of semitendinosus and gracilis tendon cross‐sectional area. Knee Surgery, Sports Traumatology, Arthroscopy, 27, 3498–3504. Available from: 10.1007/s00167-019-05421-6 30809723

[15] Holm, I. , Øiestad, B.E. , Risberg, M.A. & Aune, A.K. (2010) No difference in knee function or prevalence of osteoarthritis after reconstruction of the anterior cruciate ligament with 4‐strand hamstring autograft versus patellar tendon‐bone autograft: a randomized study with 10‐year follow‐up. The American Journal of Sports Medicine, 38, 448–454. Available from: 10.1177/0363546509350301 20097928

[16] Johnston, P.T. , Mcclelland, J.A. , Feller, J.A. & Webster, K.E. (2021) Knee muscle strength after quadriceps tendon autograft anterior cruciate ligament reconstruction: systematic review and meta‐analysis. Knee Surgery, Sports Traumatology, Arthroscopy, 29, 2918–2933. Available from: 10.1007/s00167-020-06311-y 33026536

[17] Kaplan, Y. & Witvrouw, E. (2019) When is it safe to return to sport after ACL reconstruction? Reviewing the criteria. Sports Health: A Multidisciplinary Approach, 11, 301–305. Available from: 10.1177/1941738119846502 PMC6600576 31136725

[18] Mouarbes, D. , Menetrey, J. , Marot, V. , Courtot, L. , Berard, E. & Cavaignac, E. (2019) Anterior cruciate ligament reconstruction: a systematic review and meta‐analysis of outcomes for quadriceps tendon autograft versus bone‐patellar tendon‐bone and hamstring‐tendon autografts. The American Journal of Sports Medicine, 47, 3531–3540. Available from: 10.1177/0363546518825340 30790526

[19] Paterno, M.V. , Huang, B. , Thomas, S. , Hewett, T.E. & Schmitt, L.C. (2017) Clinical factors that predict a second ACL injury after ACL reconstruction and return to sport: preliminary development of a clinical decision algorithm. Orthopaedic Journal of Sports Medicine, 5, 2325967117745279. Available from: 10.1177/2325967117745279. 29318172 PMC5753959

[20] Renström, P.A. (2013) Eight clinical conundrums relating to anterior cruciate ligament (ACL) injury in sport: recent evidence and a personal reflection. British Journal of Sports Medicine, 47, 367–372. Available from: 10.1136/bjsports-2012-091623 22942168

[21] Runer, A. , Csapo, R. , Hepperger, C. , Herbort, M. , Hoser, C. & Fink, C. (2020) Anterior cruciate ligament reconstructions with quadriceps tendon autograft result in lower graft rupture rates but similar patient‐reported outcomes as compared with hamstring tendon autograft: a comparison of 875 patients. The American Journal of Sports Medicine, 48, 2195–2204. Available from: 10.1177/0363546520931829 32667271

[22] Sajovic, M. , Strahovnik, A. , Dernovsek, M.Z. & Skaza, K. (2011) Quality of life and clinical outcome comparison of semitendinosus and gracilis tendon versus patellar tendon autografts for anterior cruciate ligament reconstruction: an 11‐year follow‐up of a randomized controlled trial. The American Journal of Sports Medicine, 39, 2161–2169. Available from: 10.1177/0363546511411702 21712483

[23] Sajovic, M. , Vengust, V. , Komadina, R. , Tavcar, R. & Skaza, K. (2006) A prospective, randomized comparison of semitendinosus and gracilis tendon versus patellar tendon autografts for anterior cruciate ligament reconstruction: five‐year follow‐up. The American Journal of Sports Medicine, 34, 1933–1940. Available from: 10.1177/0363546506290726 16923826

[24] Snaebjörnsson, T. , Hamrin Senorski, E. , Ayeni, O.R. , Alentorn‐Geli, E. , Krupic, F. , Norberg, F. et al. (2017) Graft diameter as a predictor for revision anterior cruciate ligament reconstruction and KOOS and EQ‐5D values: A cohort study from the Swedish national knee ligament register based on 2240 patients. The American Journal of Sports Medicine, 45, 2092–2097. Available from: 10.1177/0363546517704177 28460194

[25] Snaebjörnsson, T. , Svantesson, E. , Sundemo, D. , Westin, O. , Sansone, M. , Engebretsen L et al. (2019) Young age and high BMI are predictors of early revision surgery after primary anterior cruciate ligament reconstruction: a cohort study from the Swedish and Norwegian knee ligament registries based on 30,747 patients. Knee Surgery, Sports Traumatology, Arthroscopy, 27, 3583–3591. Available from: 10.1007/s00167-019-05487-2. 30879108 PMC6800860

[26] Spragg, L. , Chen, J. , Mirzayan, R. , Love, R. & Maletis, G. (2016) The effect of autologous hamstring graft diameter on the likelihood for revision of anterior cruciate ligament reconstruction. The American Journal of Sports Medicine, 44, 1475–1481. Available from: 10.1177/0363546516634011 27002103

[27] Strauss, M.J. , Miles, J.W. , Kennedy, M.L. , Dornan, G.J. , Moatshe, G. , Lind, M. et al. (2022) Full thickness quadriceps tendon grafts with bone had similar material properties to bone‐patellar tendon‐bone and a four‐strand semitendinosus grafts: a biomechanical study. Knee Surgery, Sports Traumatology, Arthroscopy, 30, 1786–1794. Available from: 10.1007/s00167-021-06738-x 34591124

